# [Corrigendum] High expression of hnRNPA1 promotes cell invasion by inducing EMT in gastric cancer

**DOI:** 10.3892/or.2026.9198

**Published:** 2026-09-21

**Authors:** Yahua Chen, Jun Liu, Wei Wang, Li Xiang, Jide Wang, Side Liu, Hongyan Zhou, Zheng Guo

Oncol Rep 39: 1693–1701, 2018; DOI: 10.3892/or.2018.6273

Subsequently to the publication of the above paper, an interested reader drew to the Editor's attention that the control GAPDH western blots shown in Fig. 1D on p. 1696 were strikingly similar to the GAPDH blots shown in Fig. 2A on p. 1697, even though the experimental conditions were reported to be different for these figures. In addition, upon analyzing the data in this paper independently in the Editorial Office, it came to light that certain of the hnRNPA1 blots shown in Fig. 1B were strikingly similar to blots that had already been published in a paper in the journal *Oncotarget* that featured two of the authors in common (Jide Wang and Side Liu).

The authors were able to consult their original data, and realized that these data had inadvertently been included incorrectly in these two figures. Revised and corrected versions of Figs. 1 and 2, now showing replacement data for the western blots featured in Figs. 1B, 1D and 2A, are shown on the next two pages. Note that the replacement of the original data with the revised data in this pair of figures has not had a significant impact on the conclusions reached in this study. The authors regret the errors that were made during the compilation of the original figures, and are grateful to the editor of *Oncology Reports* for allowing them the opportunity to publish this corrigendum. All the authors agree with the publication of this corrigendum; furthermore, they apologize to the readership for any inconvenience caused.

## Figures and Tables

**Figure 1.**
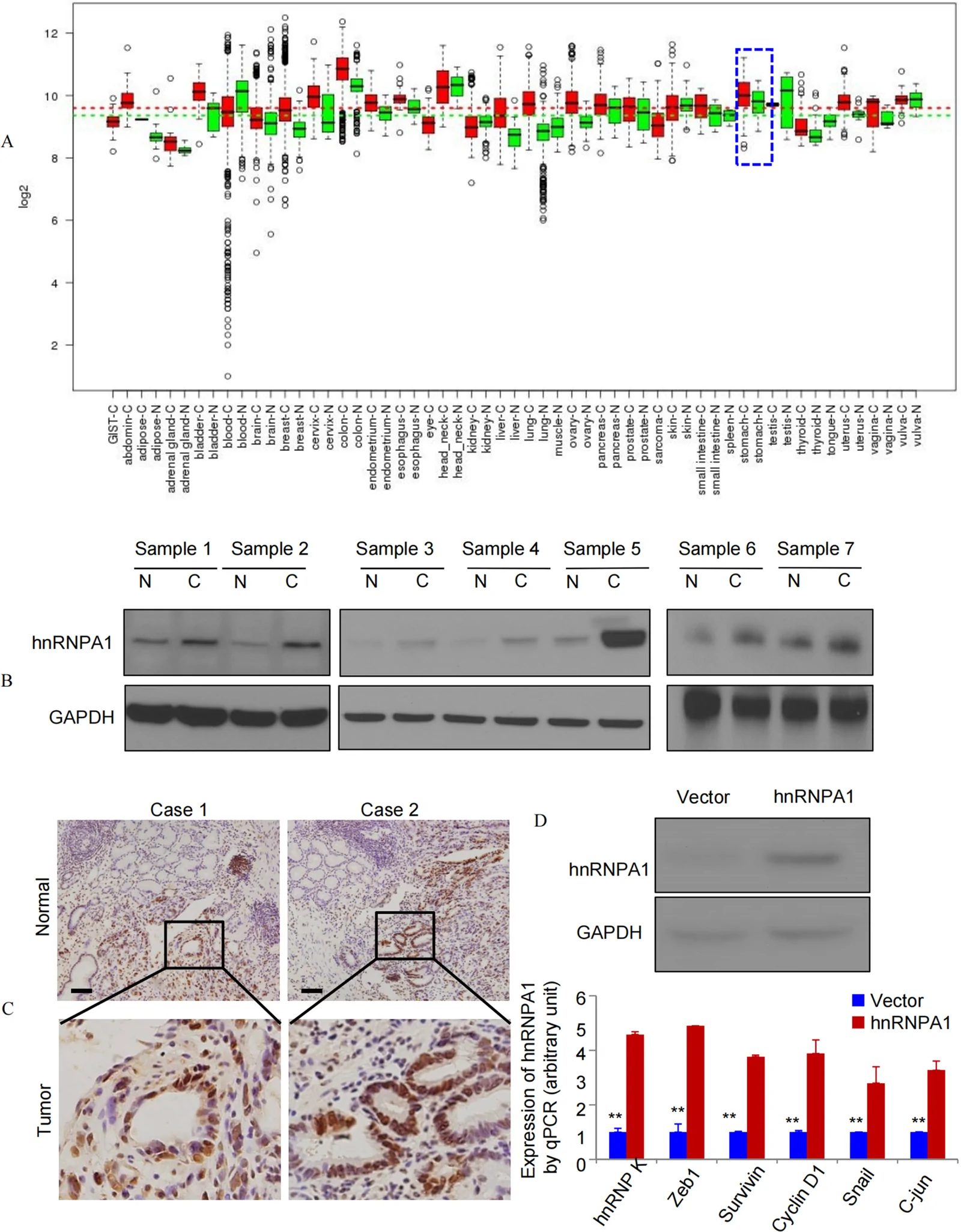
HnRNPA1 expression in GC is higher than in normal cells. (A) GENT database was used to find the hnRNPA1 mRNA expression in various types of cancer. Boxes represent the median and 25th and 75th percentiles, dots represent outliers, red boxes represent tumor tissues, green boxes represent normal tissues and red and green dashed lines represent the average value of all tumor and normal tissues, respectively. HnRNPA1 mRNA expression in gastric tissue, represented by blue dotted lines. (B) Resected tumors and adjacent non-tumor tissue specimens were lysed and hnRNPA1 protein expression was detected by western blot analysis. C, gastric carcinoma; N, normal gastric tissues (n=7). (C) In two selected patient cases, higher expression of hnRNPA1 in tumor tissues was confirmed by IHC. Scale bars, 100 µm. (D) Western blotting was used to access the expression of hnRNPA1 in stable transfectants. (E) The expression of multiple oncogenes in vector and hnRNPA1 stable cells was detected by qPCR. **P<0.05.

**Figure 2.**
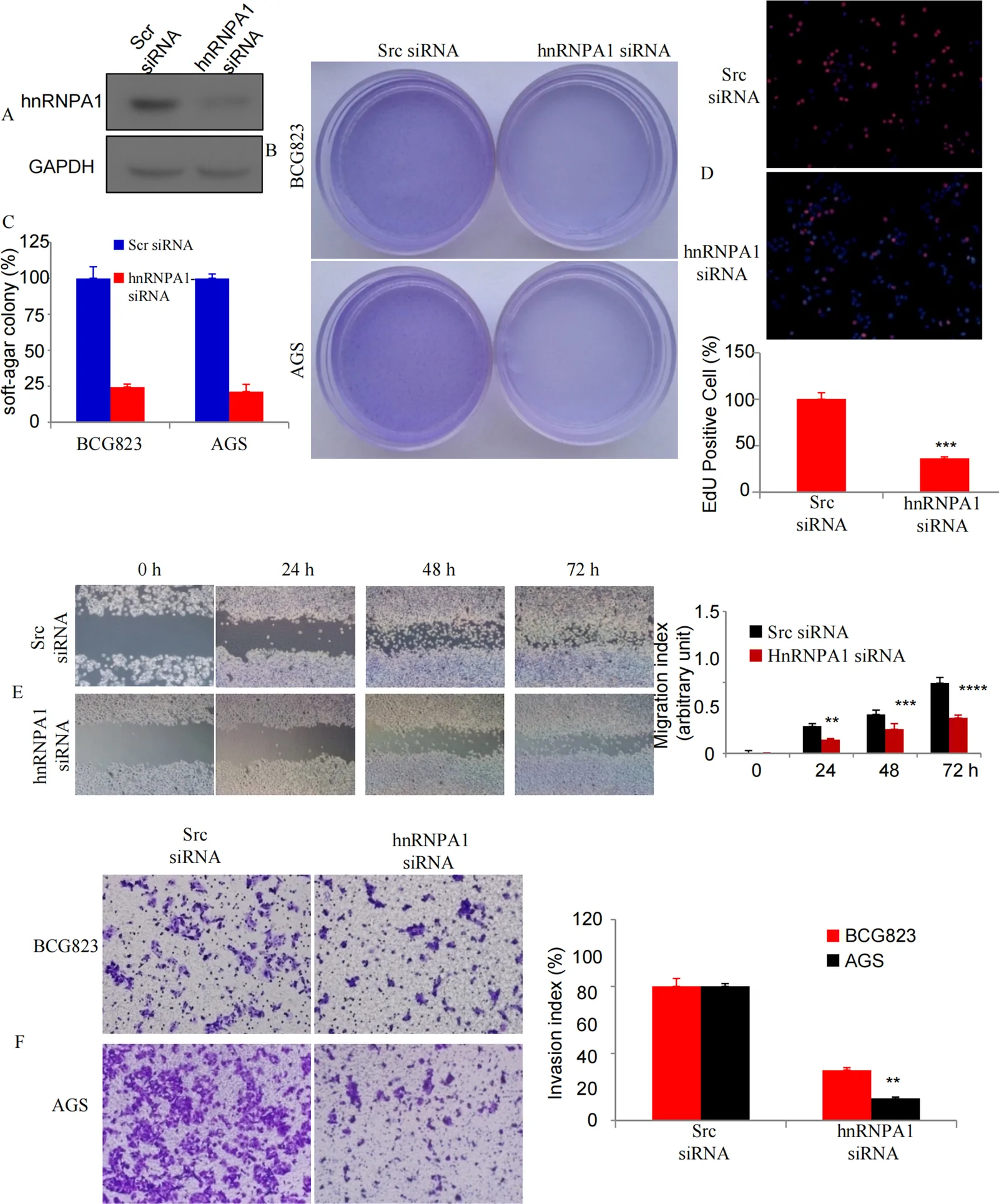
HnRNPA1 facilitates the malignant biological behavior of GC cells. (A) HnRNPA1 expression was detected by western blot analysis in BCG823 and AGS cells. (B and C) HnRNPA1-silenced GC cells and scr siRNA cells were plated individually in soft agar culture dishes with complete culture medium. Cell colonies were visualized after 12 days. *P<0.05. (D) DNA synthesis of the BCG823 cells was assessed using an EdU incorporation assay after transfection at 48 h. ***P<0.01, between hnRNPA1 and Scr siRNA. (E) Monolayer BCG823 cells transfected with the indicated siRNA were wounded and images at different time-points were captured. **P<0.05, ***P<0.01, ****P<0.001. (F) The invasive potential of the BCG823 and AGS cells transfected with Scr siRNA or hnRNPA1 siRNA was evaluated by invasion chamber and assessed by invasion index. **P<0.05. These experiments were repeated at least three times.

